# Identification of symptom domains in ulcerative colitis that occur frequently during flares and are responsive to changes in disease activity

**DOI:** 10.1186/1477-7525-6-69

**Published:** 2008-09-20

**Authors:** Joel C Joyce, Akbar K Waljee, Tahira Khan, Patricia A Wren, Maneesh Dave, Ellen M Zimmermann, Sijian Wang, Ji Zhu, Peter DR Higgins

**Affiliations:** 1 Medical School, University of Michigan, Ann Arbor, MI, USA; 2 Division of Gastroenterology, Department of Internal Medicine, University of Michigan, Ann Arbor, MI, USA; 3 School of Health Sciences, Oakland University, Rochester, MI, USA; 4 School of Public Health, University of Michigan, Ann Arbor, MI, USA; 5 Department of Statistics, University of Michigan, Ann Arbor, MI, USA

## Abstract

**Background:**

Ulcerative colitis disease activity is determined by measuring symptoms and signs. Our aim was to determine which symptom domains are frequent and responsive to change in the evaluation of disease activity, which are those defined by three criteria: 1) they occur frequently during flares; 2) they improve during effective therapy for ulcerative colitis; and 3) they resolve during remission.

**Methods:**

Twenty-eight symptom domains, 16 from standard indices and 12 novel domains identified by ulcerative colitis patient focus groups, were evaluated. Sixty subjects with ulcerative colitis were surveyed, rating each symptom on the three criteria with a 100 mm Visual Analogue Scale. Frequent and responsive symptoms were defined *a priori *as those whose median Visual Analogue Scale rating for all 3 criteria was significantly greater than 50.

**Results:**

Thirteen of the 28 symptom domains were identified as both frequent in ulcerative colitis flares and responsive to changes in disease activity. Seven of these 13 symptom domains were novel symptoms derived from ulcerative colitis patient focus groups including stool mucus, tenesmus, fatigue, rapid postprandial bowel movements, and inability to differentiate liquid or gas from solid stool when rectal urgency occurs. Ten of the 16 symptom domains from standard indices were either infrequent or unresponsive to changes in disease activity.

**Conclusion:**

Only some of the symptoms of ulcerative colitis that are important to patients are included in standard indices, and several symptoms currently measured are not frequent or responsive to change in ulcerative colitis patients. Development of survey measures of these symptom domains could significantly improve the assessment of disease activity in ulcerative colitis.

## Background

Ulcerative colitis (UC) is a chronic inflammatory disease that affects more than 600,000 Americans [1]. Controlling inflammation and therefore symptoms are the primary goals of treatment, but current therapies are only moderately effective, as several population-based studies have demonstrated that patients with UC have a 9–24% ten year colectomy rate [2-4] and a 33–45% twenty-five year cumulative colectomy rate [5,6]. Many potential new therapies for ulcerative colitis are currently being developed in preclinical testing and clinical trials, including molecules targeting interleukin 12, interleukin 17, and endothelial integrins.

The efficacy of UC therapies in clinical trials is assessed with disease activity indices that typically combine clinical symptoms, physician assessment, and endoscopy to measure severity [7,8]. A large number of indices have been developed over the past 50 years that attempt to measure disease activity in ulcerative colitis, recently summarized and reviewed in D'Haens *et al *[9]. These include the Mayo Index [7], the UCDAI [8], the Seo Index [10], the Ulcerative Colitis Clinical Score [11], the Simple Clinical Colitis Activity Index [12], and the St. Mark's Index [13]. However, D'Haens *et al *notes that there has been "considerable heterogeneity and confusion regarding the optimal instruments (activity indices) and end points for assessing the efficacy of medical therapies for UC" and that "an 'optimal' scoring index for UC is still to be developed" [9]. In addition, the European Crohn's and Colitis Organization (ECCO) Consensus on UC recently stated that "Instruments for measuring clinical and/or endoscopic disease activity in UC are available, but none has been subjected to an adequate validation process" [14].

Notably, it has never been established that any of these indices actually measures all of the important components of ulcerative colitis. Most current indices were developed without patient input and items were not tested for their responsiveness to change. The lack of a patient-centered index raises the question of whether or not the available indices truly capture all of the symptoms that occur during a flare for patients with UC.

Previously, we conducted focus group interviews with UC patients who discussed their UC experience and how their symptoms related to periods of flare or remission. We were interested in capturing additional symptoms experienced by UC patients that are not currently assessed in commonly used indices. We recorded and qualitatively analyzed all signs and symptoms discussed during these group interviews and compared our findings with existing index components [15]. We concluded that current indices capture only a portion of clinical symptoms and include several symptoms not identified by patients. In addition, patients identified new symptoms not previously assessed in UC disease activity indices. We believe that current indices may not completely measure or reflect patients' experience of UC.

The lack of a patient-centered index and the numerous candidate therapies in developmental stages highlight the need to develop a new UC activity index for clinical research that is patient-centered, validated, and which will provide a rigorous benchmark for determining the clinical efficacy of new UC therapies. An ideal index for the measurement of disease activity in UC would include the symptom domains important to patients and would focus on symptoms that occur in most patients and are responsive to changes in disease activity (i.e., have a good dynamic range). Symptom domains that are responsive to changes in disease activity would reproducibly worsen during flares, improve with effective therapy, and be absent during remission. We chose to evaluate 16 symptom domains from currently existing UC disease activity indices as well as 12 novel symptom domains identified in our previous focus group study [15]. All of these 28 symptom domains were identified as important by at least one patient with ulcerative colitis in our focus groups. We decided that we would include all 28 symptom domains that were mentioned regardless of frequency in the focus groups as our further validation and testing would identify those symptoms most frequent and responsive in the experience of UC. Therefore, we aimed to determine which symptom domains would be most useful for inclusion in the development of a new patient-centered UC disease activity index by quantitatively evaluating symptom frequency and responsiveness to change in patients with ulcerative colitis.

## Methods

### Subjects

Our study was undertaken at the University of Michigan Medical Center. On a weekly basis, the University of Michigan Data Warehouse Team identified all patients with a history of ulcerative colitis who had a scheduled outpatient appointment to be seen at the University of Michigan. Patients were identified as having a documented history of UC using the ICD-9 code of 556. We also identified inpatients with UC through regular consultation with the inpatient gastroenterology service. Recruitment was performed face-to-face by a study team member either in the outpatient setting (University of Michigan Gastroenterology Clinic or endoscopy unit) or on the hospital inpatient service.

Inclusion criteria included age between 18 and 75 years, diagnosis of UC as documented in the medical record, and willingness and ability to understand and fill out the questionnaire. Exclusion criteria included a history of colectomy or past participation in this study. Our study was approved by the University of Michigan Institutional Review Board on September 20, 2006. We obtained written informed consent from all study participants prior to participation.

Basic demographic and disease characteristics were collected prior to distribution of the self-administered questionnaire. Disease location (proctitis, left-sided, pancolitis, or unknown) was determined by asking the patient if they knew the extent of their disease. Disease extent was then verified in the medical record from either a recent clinic note or colonoscopy report by the patient's gastroenterologist. Disease severity (quiescent, mild, moderate, severe, or unknown) was determined through consultation with the patient's gastroenterologist at the time of their participation in the study.

### Questionnaire

We used a self-administered questionnaire to identify symptom domains in UC that occur frequently during flares and are responsive to changes in disease severity. We defined symptom domains broadly as a symptom or sign that could be used by the patient to assess their disease activity level. Enrolled study participants were given the symptom domain questionnaire to fill out and a study team member was present to answer any questions that arose.

**Figure 1:**
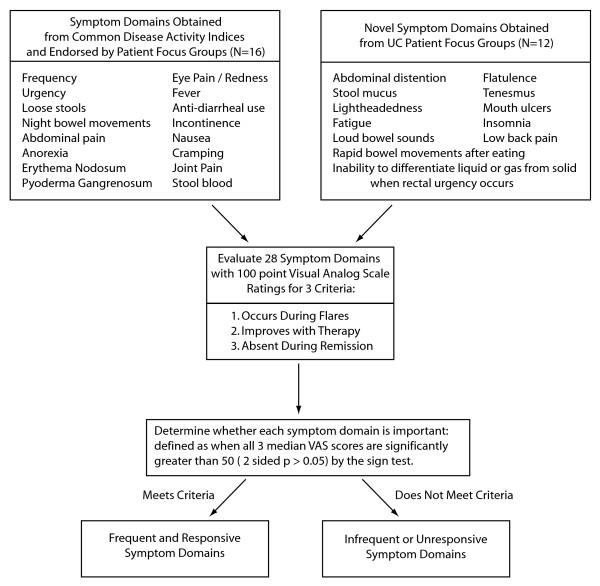
**Flow diagram of 28 symptom domains in questionnaire and criteria for determining frequent and responsive symptom domains**. Sixteen symptom domains were included from commonly used indices of ulcerative colitis disease activity (Truelove and Witts, St. Mark's Index, CAI, SCCAI, UCSS, Mayo, and UCDAI), and twelve novel symptom domains were included from previously conducted focus group input [15]. The questionnaire required ratings of the three criteria listed on a 100 mm Visual Analogue Scale for each of the 28 symptom domains. Symptom domains were determined to be either frequent and responsive or infrequent or unresponsive for evaluation of disease activity based on significance of the sign test.

**Figure 2:**
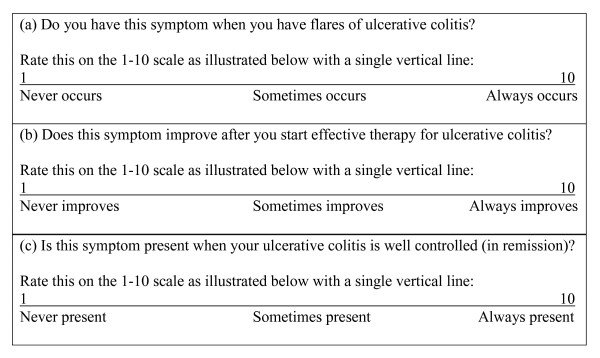
**Visual analogue scale used on questionnaire for assessing each of three endpoints for all 28 symptom domains**. An example of the 100 mm Visual Analogue Scales (VAS) used in the questionnaire to rate the three criteria for each of the 28 symptom domains.

The questionnaire consisted of three ratings of 28 symptom domains found in UC. Sixteen of these symptom domains were from standard UC disease activity indices [7,8,10-12]. The remaining 12 symptom domains were novel domains identified from focus group data [15] (Figure 1). Participants were asked to rate each of the 28 symptom domain using three separate 100 mm visual analogue scales (VAS) for each of three endpoints: 1) the symptom is present during flares; 2) if present during flares, the symptom improves with effective therapy; and 3) the symptom is absent when in remission (Figure 2). Study participants were instructed to mark with a single line at whatever point they felt best represented their experience of that symptom domain. Study participation was concluded upon successful completion of the survey questionnaire.

### Data management and statistical analysis

Two study team members (J.C.J. and T.K.) determined each symptom domain's rating for each of the three endpoints by independently measuring the distance in millimeters from the left end of the Visual Analogue Scale (0 mm) to the point where a mark was made on the VAS line. Measurements were made using the same ruler and to the nearest millimeter at the point where the mark crossed the VAS line. J.C.J. and T.K. independently entered these ratings, along with demographic information, into a Microsoft Access (Microsoft Corporation, Redmond, WA) database. The Access datasets were imported into Epi Info v. 3.3.2 (Centers for Disease Control and Prevention, Atlanta, GA) to perform error-checking. Any discrepancies in ratings for a given symptom domain endpoint were resolved by having J.C.J. and T.K. re-examine the original questionnaire and re-measure the distance of the VAS rating in contention. Consensus was reached for all measurements. The final, corrected Access dataset was imported into Stata 9.2 statistical software (Stata Corporation, College Station, TX) for analysis.

The assumption was made that study participants who assigned a rating of less than 20 mm to the first endpoint ("the symptom is present during flares") for a given symptom domain did not have that particular symptom domain present during an active flare of their UC. Therefore, it was not possible for these study participants to accurately assess the second endpoint ("if present during flares, the symptom improves with effective therapy") since therapy would not have an affect on a symptom that was not originally present during flare. For study participants in this situation, their rating for the second endpoint (improve with therapy) was dropped from the dataset, regardless of its numerical value.

As the ratings for each of the three endpoints for the 28 symptom domains were found to have skewed distributions, medians and interquartile ranges were calculated for all symptom domains for each endpoint for accurate comparison. We also performed a nonparametric sign test to determine if the medians for the endpoints were significantly greater than a cut-off of 50 mm on the Visual Analogue Scale. A two-sided p-value of < 0.05 was used to determine statistical significance for this test. We defined a frequent and responsive symptom domain as one in which ratings for all three VAS endpoints for that symptom domain are significantly (two-sided p < 0.05) greater than 50 mm (as diagrammed in Figure 1). Symptom domains in which any of the three endpoints were not significantly greater than 50 mm did not meet our criteria of a frequent and responsive symptom domain.

We expected that some symptom domains would cluster together, and might be highly correlated. In order to identify these clusters, we performed a cluster analysis and used a dendrogram to present all symptoms domains to show the correlations between the various symptom domains.

While the multiple comparisons of the 84 planned sign tests with an alpha of 0.05 would be expected to identify approximately 4 positive results (1/20) by chance, the probability of this occurring by chance in all 3 ratings of a single endpoint is 1/(20*20*20), or 1/8000. Therefore, we chose not to use an adjustment for multiple comparisons.

The required sample size was calculated on the basis of a t test to determine if the average ratings are significantly greater than 50 (null hypothesis). We assumed a sample mean of 60, and a standard deviation of 18 points on the 0–100 scale. With an alpha of 0.05, and a power of 80%, this would require a sample size of 26 subjects. We conservatively estimated that 4 subjects might fail to complete the ratings or have uninterpretable responses. As this power calculation assumes a normal distribution, and we expected skewed samples, we doubled the predicted sample size from 30 to 60 to account for non-normality of the ratings.

## Results

### Patient characteristics

A total of 60 UC patients were enrolled for participation in our study between October, 2006 and February, 2007. The demographic and disease characteristics of the enrolled study participants are presented in Table 1. The enrolled study population represented a broad range of the UC patient population at the investigators' university-based institution.

**Table 1:** Demographics and disease characteristics of patients in the study

**Characteristics**	**Participants (n = 60)**	**%**
**Gender**		
Male	31	51.7
Female	29	48.3
**Median [range] age (years)**	**39.4 [18.6–72.8]**	
**NIH Race**		
Caucasian	54	90.0
Asian or Pacific Islander	0	0.0
Black	3	5.0
American Indian/Alaskan	1	1.7
Hispanic	1	1.7
Other	1	1.7
**Median [range] disease duration (years)**	**5.0 [1–37]** **1 missing**	
**Disease location**		
Proctitis	4	6.7
Left-sided	26	43.3
Pancolitis	28	46.7
Unknown	2	3.3
**Disease Severity**		
Quiescent	3	5.0
Mild	24	40.0
Moderate	19	31.7
Severe	13	21.7
Unknown	1	1.7
**Medications**		
Current rectal therapy	14 of 60	23.3
Current steroids	24 of 60	40.0
Current oral 5-ASA's	51 of 60	85.0
Current thiopurines	15 of 60	25.0
Current infliximab	4 of 60	6.7
**Inpatient status**		
Yes	8	13.3
No	52	86.7

### Incidence of symptom domains that are frequent or responsive

**Figure 3:**
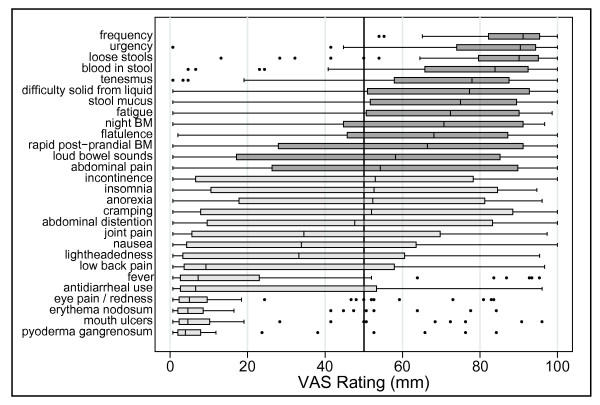
**Visual analogue scale ratings of symptoms present during a flare**. A box plot of the VAS ratings of symptoms **present during flares **for the 28 symptom domains is presented. Each box bounds the region from the 25^th ^to 75^th ^percentile of responses. The vertical line in each box is the median. Lines connect the boxes to the next observation beyond the box and the dots represent remaining outliers. The vertical line at VAS = 50 signifies the *a priori *cut-off rating establishing symptoms that are frequent during flares. The symptoms presented in dark gray boxes are those that met all 3 criteria for symptom importance. Only the 13 highest rated symptoms had VAS ratings significantly greater than 50 for presence during flares.

**Figure 4:**
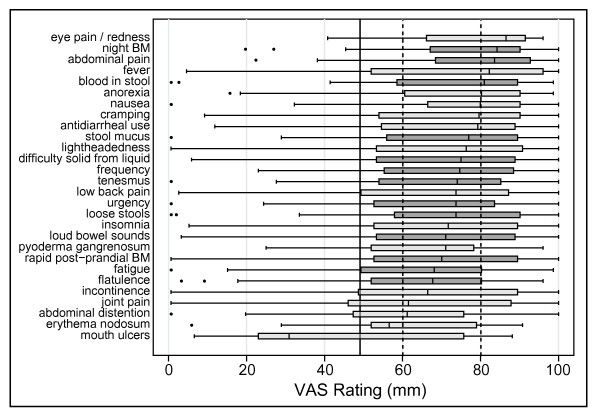
**Visual analogue scale ratings of symptoms that improve during therapy**. A box plot of the VAS ratings of symptoms is presented that **improve during therapy **All but the lowest-rated symptom, mouth ulcers, had VAS ratings significantly greater than 50 for improvement during therapy. Additional more stringent cutoffs at 60 and 80 points are presented for illustration.

Many (17 of 28) symptom domains were found to be frequently present during a UC flare, as they had a median VAS rating greater than 50 mm for our first endpoint (Figure 3). Likewise, nearly all symptoms (27 of 28, or all except for mouth ulcers) were shown to improve with therapy, as they also had a median VAS rating greater than 50 mm for the second endpoint (Figure 4). In addition, all 28 symptom domains were found to be absent in remission the majority of the time in most individuals (median VAS rating greater than 50 mm for the third endpoint) (Figure 8). However, our criteria for defining a frequent and responsive symptom domain specifically stated that all three endpoints for a particular symptom domain had to have a median VAS rating that was significantly (p < 0.05) greater than 50 mm.

**Figure 5:**
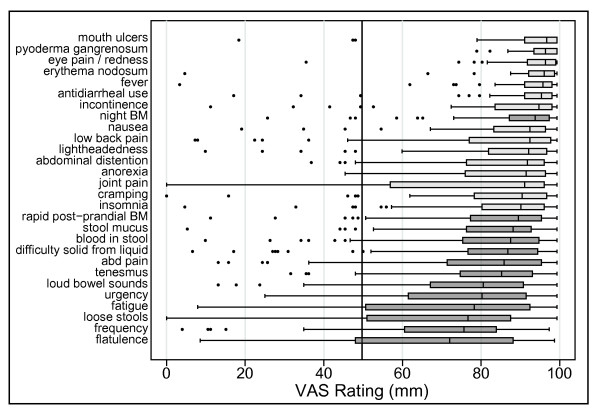
**Visual analogue scale ratings of symptoms that are absent during remission**. A box plot of the VAS ratings of symptoms that are **absent during remission**. All of the symptoms evaluated had VAS ratings significantly greater than 50 for absence during remission.

It is important to note that the decision to use 50 as the cutoff for a significant VAS rating is arbitrary. Close examination of Figure 4 reveals that several symptoms barely achieve this cutoff. If more stringent cutoffs, represented by the dashed lines at 60 and 80 points on the VAS, this would exclude 2 or 21 symptoms from further consideration in the development of a UC activity index.

### Symptoms domains that are frequent and responsive to change

Thirteen of the 28 symptom domains fulfilled the criteria of a frequent and responsive symptom domain, defined as a median VAS rating for all three endpoints significantly (p < 0.05) greater than 50 mm (left half of Table 2). Approximately half (6 of 13) of the frequent and responsive symptom domains were derived from standard indices of UC disease activity (upper left quadrant of Table 2). However, the remaining frequent and responsive symptom domains (7 of 13, 54%) were novel symptom domains elicited from previously conducted focus groups [15] that were not found in standard UC indices. These seven symptom domains are listed in the lower left quadrant of Table 2.

**Table 2:** Symptom domains and their frequency and responsiveness to changes in disease activity

	Frequent and Responsive Symptoms (n = 13)	Infrequent or Unresponsive Symptoms (n = 15)
Symptom Domains Derived from Common Indices	Loose stools (consistency) Stool blood Urgency Frequency Nighttime bowel movements Abdominal pain	Anorexia Erythema nodosum Pyoderma gangrenosum Eye redness/pain Fever Use of anti-diarrheals Incontinence Nausea Cramping Joint pain

Symptom Domains Derived from Focus Groups	Stool mucus Tenesmus Difficulty telling liquid or gas from solid stool before evacuation Rapid post-prandial bowel movements Loud bowel sounds Flatulence Fatigue	Abdominal distension Light-headedness Mouth ulcers Insomnia Low back pain

**Figure 6:**
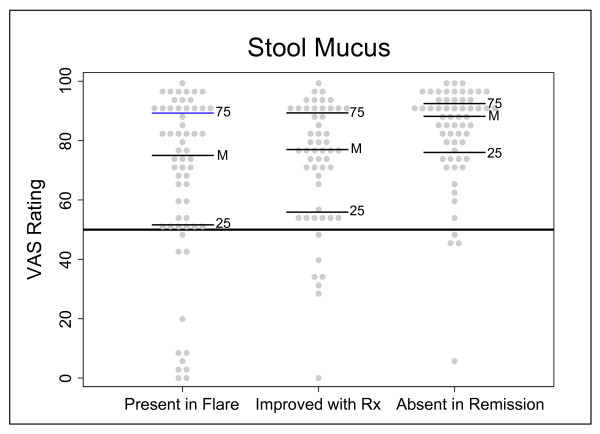
**Example of a symptom domain that meets a frequent symptom with good dynamic range**. A dot plot of a symptom domain (stool mucus) that meets criteria for a frequent and responsive symptom (one that is frequent with good dynamic range). VAS ratings are from the 100 mm scale. Each dot represents one individual's response. The horizontal lines on the graphs are as follows: 25 is the 25^th ^percentile, M is the median, and 75 is the 75^th ^percentile. The horizontal line at VAS = 50 signifies the *a priori *cut-off rating for frequent and responsive symptoms.

To illustrate the results for a representative frequent and responsive symptom domain, the findings for the symptom domain stool mucus are presented in Figure 6. In this symptom domain, 48 of 60 individuals had a VAS rating greater than 50 mm for the first endpoint ("present during flare"), 46 of 53 individuals had a VAS rating greater than 50 mm for the second endpoint ("improved with therapy"), and 56 of 60 had a VAS rating of greater than 50 mm for the third endpoint ("absent in remission"). In the case of the second endpoint for stool mucus, seven individuals were not included because their ratings for first endpoint were less than 20 mm (thus the symptom did not frequently occur in these patients – see Methods). As the median VAS rating for each of the three endpoints of the symptom domain stool mucus were all greater than 50 mm, this is a representative example of a symptom domain that fulfilled our criteria of an frequent and responsive symptom domain (frequently present and responsive to change).

### Infrequent or unresponsive symptom domains

Fifteen of the 28 symptom domains did not fulfill the criteria. These symptom domains are listed in the right half of Table 2. Noteworthy among these were 10 symptom domains commonly found in standard UC disease activity indices but are infrequent symptoms domains in our study (upper right quadrant of Table 2).

**Figure 7:**
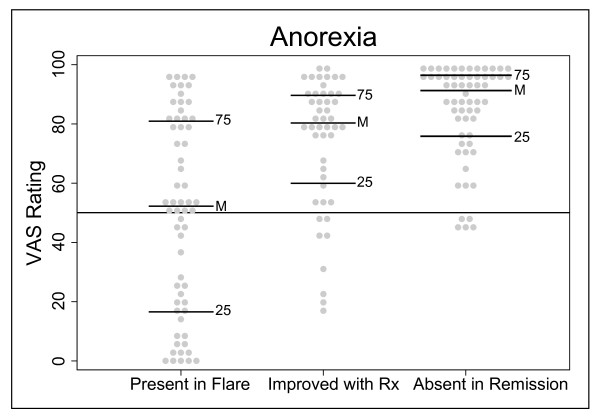
**Example of a symptom domain that did not meet criteria for a frequent symptom with good dynamic range**. Dot plot of a symptom domain (anorexia) that did not meet the criteria for a frequent and responsive symptom. VAS ratings are from the 100 mm scale. Each dot represents one individual's response. The horizontal lines on the graphs are as follows: 25 is the 25^th ^percentile, M is the median, and 75 is the 75^th ^percentile. The horizontal line at VAS = 50 signifies the *a priori *cut-off rating for frequent and responsive symptoms.

Anorexia was an example of a symptom domain that did not fulfill this study's criteria. In the case of the symptom domain anorexia, 34 of 60 individuals had a VAS rating greater than 50 mm for the first endpoint ("present during flare"), 39 of 47 individuals had a VAS rating greater than 50 mm for the second endpoint ("improved with therapy"), and 55 of 60 individuals had a VAS rating of greater than 50 mm for the third endpoint ("absent in remission") (Figure 7). As in the example with stool mucus, 13 individuals for the second endpoint in anorexia were not included because their VAS ratings for the first endpoint in anorexia were not greater than 20 mm (see Methods). As the medians of the three endpoints for the symptom domain anorexia were not all significantly greater than 50 by the sign test, this is an example of a symptom domain found on commonly used indices that did not meet our criteria and are thus classified as infrequent or unresponsive symptom domains.

**Figure 8:**
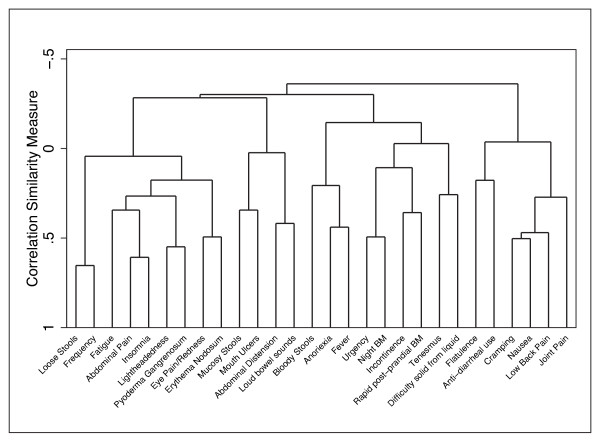
**Cluster analysis of symptom domains**. Using single clustering, a dendrogram of the 28 symptom domains is presented to illustrate the likely redundancy in some items, and the potential for item reduction in future development of a UC activity index.

## Discussion

Conventional assessment of disease activity in ulcerative colitis is done using any one of a number of disease activity indices that measure various symptoms and signs that have been deemed relevant by the designers of these indices. For the most part, items included in the various indices were determined solely by physician scientists without patient input. Therefore, it is not surprising that we found that UC disease activity indices did not include all of the symptoms that are frequent and responsive to change in UC disease activity.

We determined that several novel symptoms identified by UC patient focus groups qualify by our criteria as infrequent or unresponsive symptom domains. We also found that while several symptoms derived from current UC disease activity indices qualify as frequent and responsive symptom domains, many of the symptoms in current UC disease activity indices do not qualify. The results of this study provide us the opportunity to develop a UC disease activity index that incorporates all of the symptom domains that are frequently present during flares and are responsive to changes in disease activity.

This research study had several limitations. First, the study population was seen at a tertiary care medical center. This population may be biased towards individuals with a more severe UC disease course than is seen in the general patient population. Second, we found that individuals who did not experience a given symptom found it very difficult to assign a VAS rating to the second endpoint, "improves with therapy." We controlled for this using the method outlined (excluding improvement ratings for subjects who did not report frequently having the symptom in question). Third, as we asked patients to recollect their experience of disease flares and remissions both in the present and past, there exists the possibility of recall bias. Finally, it is possible that several of the symptoms that were identified as frequent and responsive by our criteria represent concurrent irritable bowel syndrome (IBS) symptoms, and therefore are not directly related to IBD disease activity. Post-inflammatory IBS symptoms have been shown to be relatively common in patients with IBD [16] and it is possible that IBS is part of what is being reported by participants in our study. Contrary to this view is the recent report that IBS-related symptoms in inflammatory bowel disease (IBD) correlate with increased fecal calprotectin levels, a biomarker of inflammatory activity. This suggests that symptoms typically attributed to IBS may actually indicate smoldering subclinical inflammation in IBD patients [Keohane Gastro 2007]. At this point we do not wish to exclude symptoms that may be indicative of inflammation from the evaluation of UC disease activity.

In addition, it is important to note that this study was undertaken to determine which symptom domains, as identified in our previous focus groups of UC patients [15], would be most valuable for inclusion in a new patient-centered UC disease activity index. In this study, we aimed to determine which symptom domains deserve further investigation and validation as representative of UC disease activity. This will be undertaken in development of questions for assessment of disease activity based on these frequent and responsive symptom domains and item reduction will be done through prospective testing and multivariate analysis. Validation of these questions and correlation with current disease activity indices will be undertaken in future longitudinal studies to assess for responsiveness and correlation with activity over time.

## Conclusion

The results of this study provide quantitative evidence that a subset of the additional symptoms identified in our previous work with UC patient focus groups [15] are reasonable choices for further development as part of a new index to assess UC disease activity. These additional symptoms have great value because they were identified through patient input, which allows us to capture a complete picture of disease activity in patients with ulcerative colitis. The finding that a number of symptoms used at this time in common UC disease activity indices are rarely present in UC flares and/or are less responsive to change furthers the point that by using existing measures we may not be optimally assessing a patient's UC activity. We expect that the future combination of improved survey questions for UC activity assessment and biomarkers of UC inflammation will result in better assessment of disease activity and therefore better care for patients than is possible with currently available indices.

## Competing interests

The authors declare that they have no competing interests.

## Authors' contributions

JCJ and AKW: conducted the study, analyzed and interpreted the data, and wrote the text of the article; TK: assisted in designing the study, conducted the study, and analyzed data; PAW: designed the study and contributed to writing of the text of the article; MD: conducted the study; EMZ: participated in study design and reviewed the text; SW and JZ: assisted in statistical design and interpretation of data; and PDRH: originated the idea for the study, designed the study, interpreted the data, contributed to writing of the text, and guaranteed the work. All authors have read and approved the final manuscript.
